# The Application of Magnetic Bead Selection to Investigate Interactions between the Oral Microbiota and Salivary Immunoglobulins

**DOI:** 10.1371/journal.pone.0158288

**Published:** 2016-08-02

**Authors:** Tejal Madhwani, Andrew J. McBain

**Affiliations:** Manchester Pharmacy School, The University of Manchester, Manchester, M13 9PT, United Kingdom; Oregon Health & Science University, UNITED STATES

## Abstract

The effect of humoral immunity on the composition of the oral microbiota is less intensively investigated than hygiene and diet, in part due to a lack of simple and robust systems for investigating interactions between salivary immunoglobulins and oral bacteria. Here we report the application of an *ex situ* method to investigate the specificity of salivary immunoglobulins for salivary bacteria. Saliva collected from six volunteers was separated into immunoglobulin and microbial fractions, and the microbial fractions were then directly exposed to salivary immunoglobulins of “self” and “non-self” origin. Antibody-selected bacteria were separated from their congeners using a magnetic bead system, selective for IgA or IgG isotypes. The positively selected fractions were then characterized using gel-based eubacterial-specific DNA profiling. The eubacterial profiles of positively selected fractions diverged significantly from profiles of whole salivary consortia based on volunteer (P≤ 0.001%) and immunoglobulin origin (P≤ 0.001%), but not immunoglobulin isotype (P = 0.2). DNA profiles of separated microbial fractions were significantly (p≤ 0.05) less diverse than whole salivary consortia and included oral and environmental bacteria. Consortia selected using self immunoglobulins were generally less diverse than those selected with immunoglobulins of non-self origin. Magnetic bead separation facilitated the testing of interactions between salivary antibodies and oral bacteria, showing that these interactions are specific and may reflect differences in recognition by self and non-self immunoglobulins. Further development of this system could improve understanding of the relationship between the oral microbiota and the host immune system and of mechanisms underlying the compositional stability of the oral microbiota.

## Introduction

The oral cavity is a major site where the mucosal immune system interacts with bacteria and antigens of dietary and environmental origin. The core oral microbiota reportedly maintains considerable overall compositional stability despite being an open environment [1–5]. Whilst the temporal stability of taxonomically diverse microbial communities such as the oral microbiota is likely to be mediated partly through the microbially-mediated process termed colonization resistance, other contributory factors are poorly understood (as previously reviewed [6–8]). Continual mechanical disruption of the oral microbiota, which occurs naturally and through brushing, means that nascent oral biofilms are likely to be the dominant form of microbial community in the oral cavity [9]. Since the development of dental plaque is initiated by adhesion to oral hard tissues and humoral immune components present in the saliva can variously affect adhesion to promote bacterial clearance [10], the humoral immune system is likely to play an important but currently poorly understood role in shaping the oral microbiota.

The majority of the investigations into salivary immunoglobulin recognition of resident oral microbiotas have used ELISA-based approaches where reference strains of bacteria [11–15] or oral isolates [16–19] are fixed [19], lyophilized [17] and/or extracted [12, 13, 19] for antigens to quantify immunoglobulin responses to the selected panel of bacteria. Such methods provide information about the titres of salivary immunoglobulins to the test bacterium relative to the total immunoglobulin concentrations. Whilst such approaches have contributed substantially to understanding of the interactions between oral consortia and humoral immunity, the functional significance of humoral responses to oral bacteria remains relatively poorly understood, partly due to a lack of appropriate tools to simultaneously detect responses to multiple microbes. Furthermore, applications of ELISA-based methods have been generally restricted to culturable organisms which has limited the proportion of oral bacteria that can be investigated [3, 20–22]. Here, we report the application of a magnetic bead-based method to separate components of the oral consortia that are recognized by salivary immunoglobulins, independent of culturability, in an isotype-specific manner for identification by eubacterial profiling.

## Materials and Methods

### Saliva collection and separation of bacterial and immunoglobulin fractions

Unstimulated saliva (5 ml) was collected from adult donors (n = 6) mean age 30±5 years, who did not have extant periodontal disease and had not taken antibiotics for the past 12 months prior to saliva collection. Following collection, each sample was centrifuged at 4°C, for 10 min (13, 000 x g), and separated into supernatant (antibody) and pellet (microbial) fractions. EDTA (2.0 mM) was added to the supernatant fraction to inhibit proteases [23] prior to storage as multiple aliquots of each fraction at -80°C.

### Immunoglobulin concentration evaluation in saliva samples

Concentrations of IgG and IgA in each saliva sample were quantified by ELISA using human IgA and IgG standards (10 to 100 μg.ml^-1^; Invitrogen, Paisley, UK) to obtain a standard curve. Two dilutions (1:500 and 1:1000) of each saliva sample (50 μl) were prepared in PBS (0.1M, pH 7.0) (three technical replicates) and incubated for 18 h in 96 well flat-bottomed microtitre plate (Nunc MaxiSorp, Fisher Scientific, Loughborough, UK) at 4°C. Following incubation, wells were blocked with 200 μl of 1% bovine serum albumin in PBS for 30 min followed by three washes with PBST (PBS with Tween 20 at 0.1% v/v). Bound antibody was probed with 50 μl of 1:10,000 (in PBST) biotinylated (goat) anti-human IgG or IgA and incubated at RT for 45 min, followed by washing with PBST. Streptavidin-peroxidase (1:10,000 in PBST) (Roche, Hertfordshire, UK) was added to each well for 30 min and washed with PBST. O-phenyl diamine dihydrochloride (200 μl of 0.4 mg.ml^-1^) in 0.05M phosphate-citrate buffer at pH 5.0, with hydrogen peroxide (40 μl, 20% (v/v)), was added to each well and incubated in the dark for 30 min before reading at 450 nm in a PowerWave XS plate reader (BioTek Instrumentation Inc., Bedfordshire, UK). All saliva samples were diluted before the positive selection procedure to give equal concentrations of IgA and IgG in each sample.

### Preparation of isotype specific immunomagnetic beads

**Fig 1:**
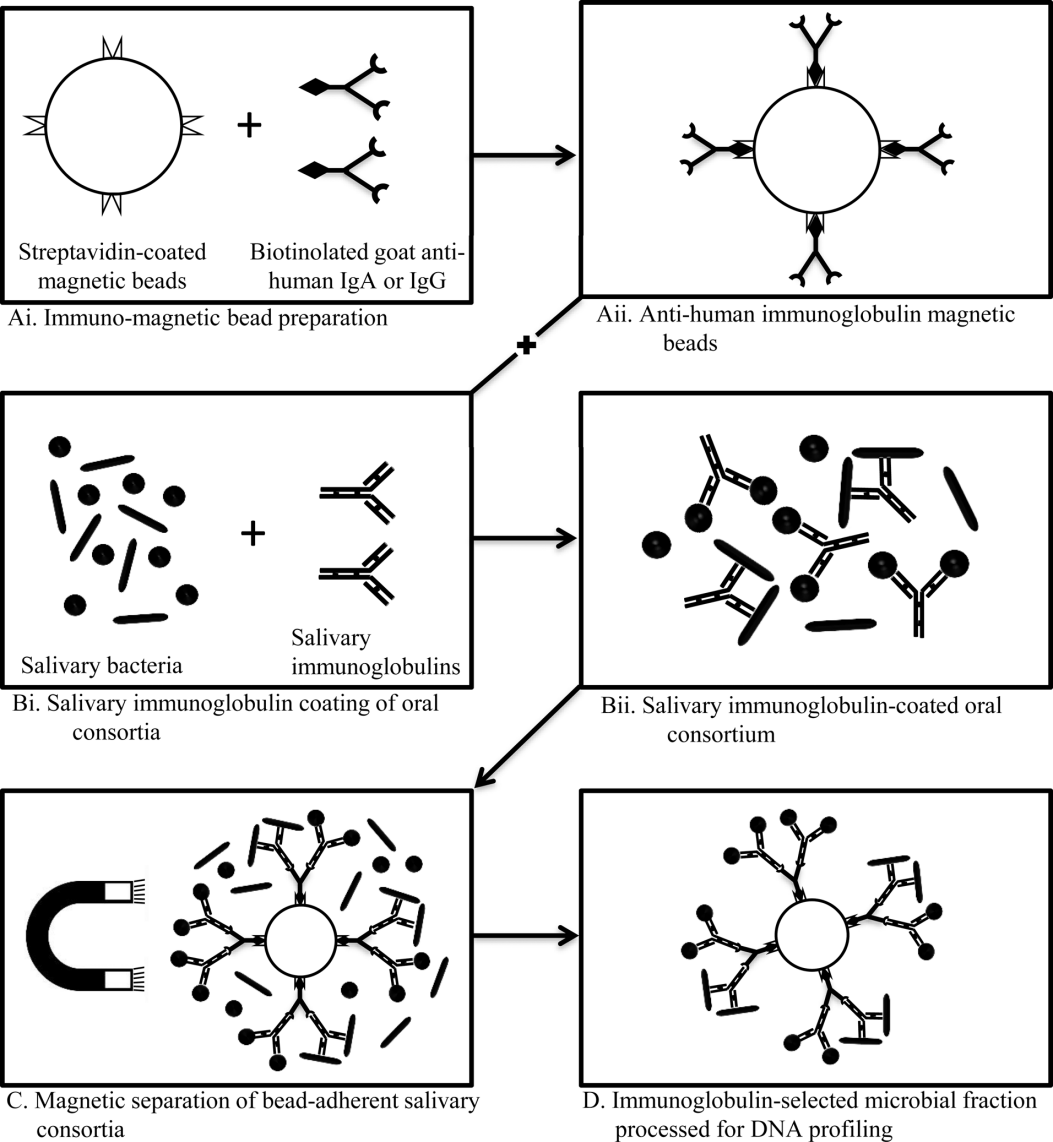
Schematic representation of the procedure for separation of salivary immunoglobulin recognized salivary consortia. (A) preparation of isotype specific magnetic beads; (B) coating of salivary consortia with immunoglobulins; (C) separation of immunoglobulin-recognized microbial consortia from complete oral consortium using magnetic bead separation; and (D) magnetic bead-adherent bacteria remaining after washing the non-adherent fraction.

Magnetic selection beads were prepared by coating biotinylated-anti human IgA or anti-human IgG (Sigma-Aldrich, USA) on MyOne Streptavidin T1 Dynabeads^®^ (Invitrogen Dynal AS, Oslo, Norway). Briefly, 10 μl of beads were washed 3x in 50 μl PBS with 0.1% BSA (PBS-BSA) to remove preservatives, and resuspended in 10μl PBS-BSA. Beads were incubated with 90 μl of 1:10,000 biotinylated anti-human IgA/G (at a concentration higher than the saturation concentration of the beads) for 30min at room temperature on an orbital shaker. Excess loosely bound immunoglobulins were eliminated by washing with 50 μl PBS-BSA and 50 μl PBST, each three times to remove free biotinylated immunoglobulin in the bead suspension and obtain anti-human immunoglobulin coated magnetic beads (Fig 1(A)).

### Magnetic bead-based separation of immunoglobulin-recognized salivary bacteria microbiotas

Salivary bacteria, collected as described above, were exposed to self and non-self immunoglobulins (n = 2), each in a separate tube, to allow immunoglobulin binding on microbiotas recognised by them (18 combinations in total) (Fig 1Bi). The reaction mixtures were prepared in duplicate (n = 36), a set each for IgA and IgG. In order to ensure that results across samples were comparable, the concentration of immunoglobulins and microbial counts of each source microbiotas were measured and standardized among samples. Salivary antibody suspension (20 μl), diluted appropriately to the same IgA or IgG concentration was added to 1 ml of the bacterial suspension (adjusted to 10^6^ Log_10_ CFU.ml^-1^) in PBS-BSA as a block, and incubated for 30 min at room temperature with gentle shaking to allow for antibody coating of bacteria. The microbiota-antibody suspension was then washed 3 x in 50 μl PBS using repeated resuspension and centrifugation, to remove unbound salivary immunoglobulins and resuspended in 1 ml of PBS. Anti-human immunoglobulin IgA/IgG-coated MyOne Streptavidin T1 Dynabeads^®^ (Invitrogen Dynal AS, Oslo, Norway) beads in PBS-BSA (10μl) were added to each selection mixture and incubated for 30 min at room temperature (Fig 1C). Each mixture was then placed in the proximity of a magnet for no more than 5 min and unbound suspension was aspirated. The bead-bound fraction held in place by magnetic beads was washed three times with 50 μl PBS-BSA and 50 μl PBST to remove loosely bound cells. The flow-through was discarded yielding the salivary immunoglobulin selected fraction (Fig 1D). This final positively selected fraction was resuspended in 100 μl PBS from which 10 μl was pooled, vortexed for 3 min and immediately plated onto blood agar (n = 3), and incubated at 37°C, for 1 week in anaerobic conditions (gas mix: 90% N2, 10% CO_2_ and 10% H_2_) within an MG1000 anaerobic workstation (Don Whitley Scientific, Shipley, UK). Unique colonies were selected for taxonomic profiling by eubacterial 16S rRNA-specific PCR DGGE. Each of the fractions (remaining 90 μl) was individually eluted from the beads by heating at 90°C for 5 min. The supernatant after centrifugation to remove the beads and cell debris was used as the DNA template representing the immunoglobulin-selected oral consortia and was stored at -80°C for future use as DNA template for eubacterial profiling. Blocking antibodies i.e. rabbit anti-human IgA and anti-human IgG at a 1:10,000 dilution in 100 μl saliva (pooled) were used to bind salivary antibodies that were then used for bacterial separation (pooled) using the same procedure described and subjected to eubacterial profiling procedure for assessment of assay validity.

### Eubacterial DNA profiling

PCR amplification, DNA profile generation by DGGE and visualisation/band identification was performed as published previously [24]. Briefly, DNA was individually extracted from the complete, unseparated salivary microbiotas of the six volunteers, as well as salivary immunoglobulin-recognized microbial fractions, and used as template for amplification of the V2-V3 region of 16S rRNA by HDA primers [25] and resolved on polyacrylamide DGGE gels. Following staining, profiles were recorded with a Canon DSLR camera (Canon, Surrey, UK) and major recurring bands on DGGE gel were excised and suspended in PCR water which was used as template for amplification of partial 16S rRNA gene by HDA2 primer. For bacterial colonies, DNA was extracted from two colonies by boiling for 10min and centrifuging at 13,000 rpm for 10 min to remove cell debris. The supernatant was then used as a DNA template for partial eubacterial (800 bp) PCR reaction. Each PCR reaction mixture consisted of 8FPL1 (5'-GAG TTT GAT CCT GGC TCA G-3'); [26] and 806R (5'-GGA CTA CCA GGG TAT CTA AT-3') primers [27]. Resulting sequencing electropherograms were verified in CHROMAS-LITE (Technelysium Pty. Ltd., Brisbane, Australia) and sequence homologies were retrieved by BLAST from EMBL database.

### Analyses of DNA profiles

Negative gel images of microbial profiles obtained above were merged using Adobe Photoshop Elements 7.0 (Adobe, London, UK). Microbial profiles were compared by generation of similarity matrices in Bionumerics v.5.1 (Applied Maths, Sint-Martens-Latem, Belgium). Here, inter-gel alignment for accurate transferability of band positions was guided by use of internal standards as well as confirming identities of recurring bands in different lanes.

### Statistical analyses

Eubacterial diversity within PCR-DGGE consortial profiles was assessed using the Shannon-Weaver index of diversity (H'); [28, 29] according to the following equation where *“s”* is the total number of species (species richness) and “p_*i*_” is the proportion of individuals belonging to the *i*^th^ species in the sample. The Mann-Whitney U test was then performed on selected samples using SPSS version 11.5 (Chicago, Illinois, USA).

$$H^{\prime }=-\sum_{i-1}^{s}p_{i}\;\ln\;p_{i}$$

### DNA profile analyses

Underlying band class data tested to evaluate the impact of salivary consortium source, salivary immunoglobulin origin and immunoglobulin isotype on the microbial selection profiles in PRIMER (v. 6) (Primer-E Ltd., Lutton, United Kingdom) as previously outlined [24]. Briefly, hierarchical clusters were generated from Bray-Curtis similarity values for the band class data. Each microbial community was reduced to two dimensions by non-metric multi-dimensional scaling (MDS) and the statistically significant clusters were identified by similarity profile permutation test. Analysis of similarity (ANOSIM) test was applied to these clusters to further identify the parameter that significantly influenced the clustering.

### Ethics and consent

Advice was taken from the Chair of a University of Manchester Research Ethics Committee regarding the correct procedures associated with the use of human saliva samples for the *ex vivo* experiments. The committee granted exemption from formal ethics approval due to the nature of the work, but as advised, written informed consent was obtained from all volunteers and all samples were collected anonymously.

## Results

### Compositional analysis of eubacterial profiles

**Fig 2:**
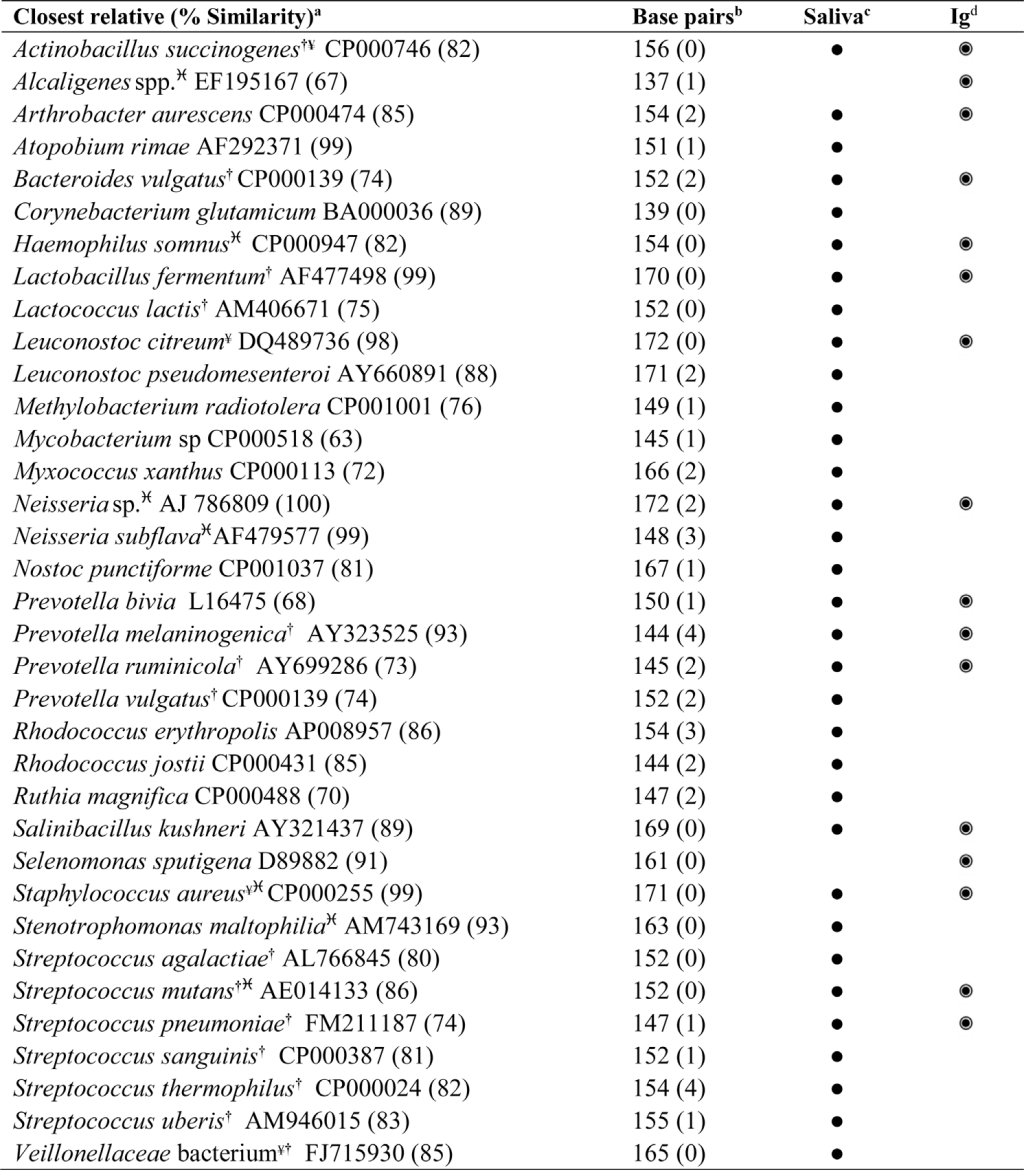
Major phylotypes identified by 16S rRNA gene sequencing from complete salivary and. Immunoglobulin-selected fractions. ^**a**^based on EMBL database searches; ^**b**^the number of ambiguous bases is given in parenthesis;. ^**c**^identified in salivary microbiota profile; ^**d**^identified in the immunoglobulin (Ig) recognized positively selected fraction; ^**†**^known oral bacterium; ^¥^multiple bands noted in same lane; ^**♓**^identified by sequencing colonies from culture of pooled positively selected fractions on blood agar.

**Fig 3:**
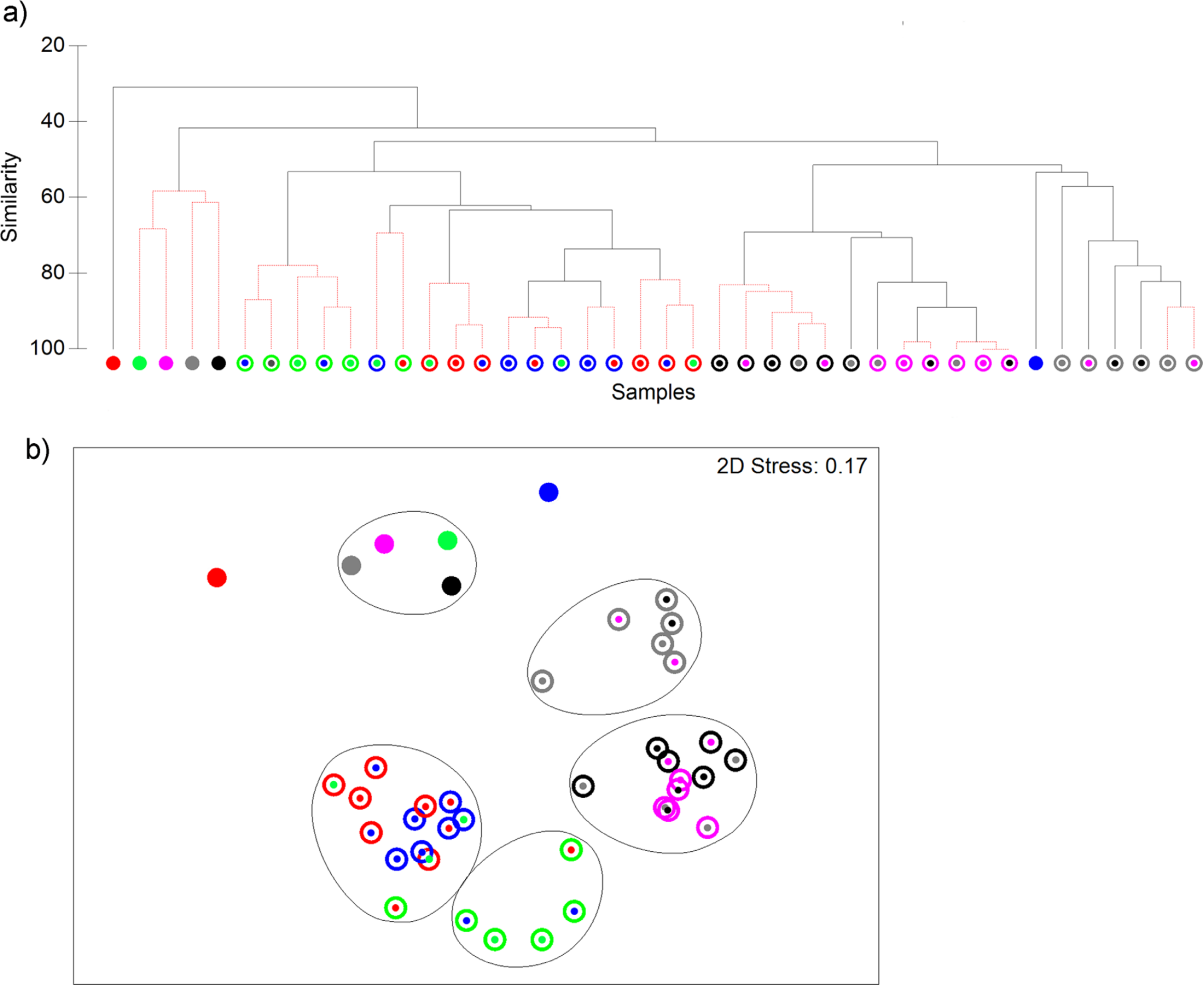
Eubacterial DNA profiles of saliva from six volunteers (indicated by red, green, pink, blue, grey and black colours) analysed by cluster analysis (a) and non-metric MDS (b). Closed circles, whole saliva, open circles, saliva eubacterial fractions selected by antibodies from volunteers as indicated by coloured centre spots. DNA profiles differed significantly between individuals for microbiota (P≤ 0.001%, R = 0.475); between microbiota selected by self vs non-self-antibodies (P≤ 0.001, R = 0.42); according to microbiota and immunoglobulin origin (P≤ 0.001, R = 0.63), but not immunoglobulin isotype (p = 0.2, R = 0.029). Robustness of clusters (p≤ 0.05) is indicted in red, based on the SIMPROF test in PRIMER v6. Contour lines on the MDS plot superimpose 55% resemblance levels derived from cluster analysis.

In order to verify the specificity of bacterial selection, anti-IgA and IgG antibodies were used to block immunoglobulin-microbe interactions before initiating the magnetic separation. Amplification of 16S rRNA genes from the magnetically separated fractions, obtained after use of the blocking antibody, yielded no detectable DNA, suggesting that the selections were due to specific interaction with antibodies. Following magnetic bead separation, all eubacterial profiles of antibody-selected fractions exhibited lower diversity compared to complete salivary consortia as evidenced by Shannon-Weiner diversity indices of DGGE profiles (H′ = 3.22 ± 0.14 and 3.54 ± 0.29, respectively) as well as by eubacterial profile analysis (Figs 2 and 3). In order to determine the identity of bacteria associated with positively selected fractions, bands were excised to include: i) bands recurring in all positively selected fractions; ii) bands present in complete salivary consortia but not present in the salivary selections; and iii) differences between selections by “self” and ‘non-self’ immunoglobulins. The summary of all identified bands have been enlisted in Fig 2. The major recurring bands in all positively selected fractions included *Lactobacillus fermentum* (AF477498), *Bacteroides vulgatus* (CP000139) *and Haemophilus somnus* (CP000947), as well as environmental organisms including *Arthrobacter aurescens* (CP000474), *Salinibacillus kushneri* (AY321437) and *Leuconostoc citreum* (DQ489736). Bands observed in the whole salivary fractions but not the positively selected fractions are evident from the comparison of saliva and Ig columns in Fig 2. Separations performed by “self” immunoglobulins differed from those using “non-self” immunoglobulins by loss of bands representing bacteria phylogenetically related to *Selenomonas sputigena* (D89882), *Staphylococcus aureus* (CP000255), *Lactobacillus fermentum* (AF477498), *Leuconostoc citreum* (DQ489736), *Streptococcus agalactiae* (AL766845) and *P*. *ruminicola* (AY699286). Furthermore, majority of the bacteria identified by sequencing colonies isolated by culture of selection fractions (except *Neisseria subflava* (AF479577) and *Alcaligenes* spp. (EF195167) were those detected in excised bands from DGGE gel of the salivary and positively selected fractions (Fig 2).

### Statistical analyses of eubacterial profiles

Analysis of eubacterial profiles by hierarchical clustering (Fig 3) identified compositional differences between complete salivary consortia and their respective positively selected fractions ranging from 30% to 50%. According to the non-metric MDS analyses, >55% similarity was observed amongst immunoglobulin-recognized profiles as well as for whole salivary consortia. For intra-cluster analysis, the ANOSIM test was applied to highlight the variables that influenced the positively selected profiles. This indicated that there were significant differences between eubacterial profiles of salivary consortia obtained from different hosts (p≤0.001). Moreover, significant differences in immunoglobulin-selected profiles were observed with use of different host consortia as well as immunoglobulin origin, self and non-self (p≤0.001); but not isotype (p = 0.2).

## Discussion

The oral microbiota represents a substantial antigenic load which reportedly contributes to the development of the human mucosal immune system as early as two to three weeks of age, from the early stages of colonisation (as reviewed by Tlaskalova-Hogenova *et al*. [30]). The potential influence of humoral immune components on the bacteriological composition of the oral microbiota has been previously considered by Nogueira *et al*. [31] who reported reductions in *S*. *mutans* counts in the saliva of infants concurrently with increasing titres of anti-*S*. *mutans* IgA immunoglobulins. Whilst this suggests a relationship between salivary immunoglobulins and numbers of oral bacteria, the relationship between humoral immunity and the composition of the oral microbiota as a whole remains poorly understood. With the intention of assessing the application of a method to study recognition between the oral microbiota and salivary antibodies, we have used a modified magnetic bead-based cell separation technique to capture salivary immunoglobulin-coated bacteria from the oral microbiota. Salivary microbiotas from six volunteers were used as target consortia to evaluate applicability of this technique for detecting differences in magnetically separated microbial consortia by different immunoglobulin origins and isotypes. Immunoglobulins of self and non-self-origin were used as representatives of humoral responses to distinct oral consortia; and IgA and IgG isotypes, which represent two developmentally and functionally distinct immunoglobulin classes.

Data indicate that the method enabled separation of a less diverse subset of the oral microbiota, as evidenced by lower diversity scores in magnetic-bead selection DNA profiles (Fig 2); lower (p<0.05) mean diversity index (H′) and less than 55% mean similarity with the whole salivary consortium in cluster analysis (Fig 3). The selected microbiotas differed from the source consortia and in comparison to each other, based on the donor microbiota (p≤0.001; Fig 3). This reflects inter-individual differences observed between oral consortia of individuals (Fig 3) [20–22].

Further statistical assessment by MDS coupled with ANOSIM, comparing selections according to immunoglobulin origin *i*.*e*. self-versus non-self, and isotypes *i*.*e*. IgA versus IgG showed statistically significant (p<0.001) compositional differences between selections by self versus non-self immunoglobulins (Fig 3). However, no statistically significant differences were observed when selections with different immunoglobulin isotypes were compared.

The apparent lack of difference between IgA and IgG isotype selection is likely to be a biological effect more than an artefact. Immunization studies suggest concurrence of IgA and IgG responses. In one such study, mice were immunised with either wild type *S*. *gordonii* or *S*. *gordonii* engineered to express an *S*. *pyogenes* surface antigen and both groups were reported to have increased titres of salivary IgA and IgG to the respective strains, although the responses were higher with the engineered strain [32]. Moreover, the possibility of similarity between IgA and IgG responses being due to non-specific bead-bacterium interactions has been precluded by the lack of 16S rRNA amplicons from selections performed after using salivary immunoglobulin blocking antibodies. Therefore, despite lack of difference between IgA and IgG responses does not prevent the technique from being used for IgA and IgG specific investigations.

Sequencing of salivary consortia revealed the presence of oral bacteria as well as putatively transient organisms. The majority of the organisms identified by culture were also identified by eubacterial DNA profiling of whole saliva, except alcaligenes (Fig 2), which was identified by culture but not DGGE. Since culture may enrich particular culturable organisms, the detection threshold may be lower compared to the mixed species direct DNA analyses. Overall, this technique has demonstrated substantial promise in terms of sensitivity. However, the technique is likely to select bacteria targeted by high abundance immunoglobulins.

In conclusion, we report the application of a simple and reasonably rapid *ex situ* technique that facilitates the selection and identification of salivary immunoglobulin-recognised bacteria. Further application of the technique may contribute towards a better understanding of host-microbe interactions involved in the maintenance of microbial stability in the oral cavity.
